# Entanglements of loneliness and mental ill health among young adult women

**DOI:** 10.1080/17482631.2020.1838101

**Published:** 2020-11-01

**Authors:** Anna Reetta Rönkä, Vappu Sunnari, Anja Taanila

**Affiliations:** a History of Sciences and Ideas, University of Oulu, Oulu, Finland; b Gender Studies, University of Oulu, Oulu, Finland; c Center for Life Course Health Research, University of Oulu, Oulu, Finland

**Keywords:** Loneliness, young adults, girls, women, mental health disorders, social issues, violence

## Abstract

**Purpose:**

If a person experiences both loneliness and mental ill health, it may have severe effects on a person’s wellbeing and functioning. This study explored the ways in which loneliness may be entangled with mental ill health and the factors that contribute to the development of such entanglements.

**Methods:**

The study participants were women from the Northern Finland Birth Cohort 1986 study, who, in 2001–2002, responded to the survey about being very lonely at the age of 15–16. Women (n = 17) were interviewed again at age 27–28 with semi-structured interviews. Data was analysed with thematic analysis.

**Results:**

Loneliness and mental ill health formed four types of entanglements: Entanglement 1 (E1): “Loneliness periodically evoked depressive feelings”; E2: “Loneliness and depressive feelings formed an ongoing spiral”; E3: “Loneliness and diagnosed depression/serious emotional disturbance formed periodic spirals;” and E4: “Serious emotional disturbance/mental illness and loneliness formed an ongoing, difficult spiral.” Social adversities like unsafe family environment, lack of close friends, and school violence contributed to the development of Entanglements 1–3. In Entanglement 4, serious mental health disorder caused loneliness.

**Conclusions:**

Loneliness was centrally linked to mental health issues in the present study and should be taken into consideration when providing mental health prevention and treatment.

## Introduction

Humans are social beings. As such, positive, reciprocal, and meaningful social relationships are at the core of our well-being. They have a positive influence on our mental and physical health (Mushtaq et al., 2014; Pandeya, 2017). However, loneliness—a common, negative, and involuntary experience—can impede this. According to previous studies, 70 to 80% of children and adolescents are familiar with this experience (Berguno et al., 2004; Galanaki & Vassilopoulou, 2007), while 5–10% experience severe, chronic, and ongoing loneliness (Asher & Paquette, 2003; Junttila, 2010; Qualter et al., 2015). National surveys in Finland show that, about 20% of people in different age groups, ranging from age eight (Lempinen et al., 2018) to the adult population between ages 20 and 70, experience some level of loneliness (Murto et al., 2015). Furthermore, earlier studies have shown that girls and women report more loneliness in comparison to boys^1^ (Kämppi et al., 2012; Rich Madsen et al., 2016; Rönkä, 2017; Rönkä et al., 2014; Stickley & Koyanagi, 2016). For instance, in a recent survey conducted among girls in the eighth and ninth grades of compulsory school in Finland, 15%reported experiencing loneliness quite often or constantly, while 6% of boys reported this experience (Finnish Institute of Health and Welfare, 2019).^2^

### Defining loneliness

Loneliness is a multidimensional experience. It typically emerges when a person experiences a need for and lack of social relationships in terms of quantity (number of social others) and/or quality (i.e., the closeness, meaningfulness, and “depth” of relationships) (Peplau & Perlman, 1982; Weiss, 1973). Following this, loneliness has been commonly divided into two types on the basis of their social sources: *loneliness of social isolation* and *loneliness of emotional isolation* (Jobe-Shields et al., 2011; Junttila & Vauras, 2009; Victor et al., 2005; Weiss, 1973). Loneliness of social isolation refers to a lack of groups or networks of other people with common interests and activities (Weiss, 1973). For example, a person may have a partner or close best friend but may still experience loneliness of social isolation, at least occasionally, as she or he experiences a social need to belong to a group (Weiss, 1973). With loneliness of emotional isolation, a person is longing for close, emotionally meaningful, reciprocal, and intimate relationships. A person with a more serious type of loneliness may experience both forms of isolation. Overall, loneliness is a subjective experience, and it is difficult to externally determine who is experiencing it (Coyle & Dugan, 2012).

Besides being subjective and relational, there are other dimensions of loneliness that influence its emergence among young people (Hawkley et al., 2008; Rönkä, 2017). Situational factors, such as relocation, may contribute to loneliness, as it may be challenging to form new social relationships in a new context (Rönkä et al., 2018; Weiss, 1973). Moreover, young people from rural or remote areas who do not relocate may experience loneliness if many of their peers migrate (Bjerk Bennike et al., 2016; Jobe-Shields et al., 2011).

Furthermore, different norms and normativities may influence loneliness. First, social norms may affect what is considered *an adequate level* or *quality* of social relationships for a person in a certain sociocultural context. According to the cognitive theory of loneliness, a person may conduct social comparisons between their existing social relationships and those of other people and begin to perceive their relationships as inadequate (De Jong Gierveld, 1998). In turn, especially gender researchers have focused on how norms and normativities might construct and maintain gendered prejudices, biases, divisions, and exclusions in different contexts (Butler, 1990, 1993, 2015; Cohen, 2013). Moreover, in our earlier interview study of 27–28-year-old young adults, we observed that *(hetero)gender(ed) norms* and *heteronormativity* seemed to intensify experiences of loneliness and a sense of not belonging among young people in Northern Finland (Rönkä et al., 2018). Gender(ed) norms regulated what adolescent girls’ and boys’ interests should have been and the “allowed” and “accepted” ways of “performing” masculinity and femininity for boys and girls, respectively. However, not following these cultural gender norms and their local and situational manifestations contributed to the development of a trajectory of more difficult and intense loneliness from childhood to young adulthood. Later, in young adulthood, when culturally assigned, (hetero)normative paths—such as forming a heterosexual marriage and having children at a “culturally appropriate” age, which in Finland is around 30 years of age—were no followed, there seemed to be an intensification of loneliness (Rönkä et al., 2018). Indeed, young adults especially are at a very important and often challenging stage in their lives, as they are transitioning towards adulthood, and many aspects of their lives are in flux, including education, work, and social relationships (Arnett et al., 2014). Different kinds of major life changes may also have an effect on loneliness experiences within this age group.

Finally, prolonged loneliness has been associated with many negative socioemotional, psychological, and physical health outcomes among the adolescent, young adult, and adult populations (Franssen et al., 2020; Heinrich & Gullone, 2006; Victor & Yang, 2012). This includes social anxiety (Hjeltnes et al., 2016; Maes et al., 2019), poor sleep quality and sleep problems (Mushtaq et al., 2014), hyperactivity and conduct disorders (Lempinen et al., 2018), learning difficulties (Junttila, 2015), and poor perceived self-reported health (Harris et al., 2013). Perhaps most seriously, loneliness has been associated with exacerbating suicidal behaviour (Flensborg-Madsen et al., 2012; Harris et al., 2013; Rönkä et al., 2013).

### Loneliness and depression

Associations between loneliness and mental health problems, especially depression, have been of interest in previous research on loneliness (Meltzer et al., 2013). Loneliness and depression are two distinct phenomena. While loneliness is not an “illness” and does not have specific diagnostic criteria, depression is a diagnostic “medical” condition (Beutel et al., 2017; Weeks et al., 1980). According to the commonly referenced diathesis–stress model of depression (see Stoppard, 1997), the underlying risks of depression are a combination of biological, cognitive, and psychosocial factors. Depression is also usually triggered by life stress, such as earlier and current traumas, losses, and disappointments (Stoppard, 1997).

The lifelong prevalence of mild and moderate depression is about 10 to 15% of the population in Finland, where about 6% of adolescents between 13 and 18 have a depressive disorder (Huttunen, 2016). Similar prevalence numbers have been reported elsewhere (Costello et al., 2006). Additionally, about 1% of the population experiences psychotic states (Depression: Current Care Guidelines Abstract, 2020).

The majority of earlier studies on loneliness and depression have been quantitative and cross-sectional (Cacioppo et al., 2006; Djukanovic et al., 2015). Many studies have found statistically significant *associations* or *correlations* between loneliness and depression in different age groups (Beutel et al., 2017; Page et al., 2010; Qualter et al., 2013; Russell et al., 1978; Rönkä et al., 2014; Shevlin et al., 2013). These findings allow more than one interpretation of the “direction” of causality between the explored variables. They also show that loneliness can be a risk factor for depression and that depressed people may isolate themselves to reduce the stresses of life (Dill & Anderson, 1999; Meltzer et al., 2013). Signs of reciprocal simultaneous causality have also been detected in the literature (Luo et al., 2012).

Moreover, and as mentioned earlier, loneliness, like depression, might be especially strongly experienced in certain life stages. Recently, the period between ages 18 to about 29 has been identified as *emerging adulthood*, referring to a developmental and life stage between that of adolescence and young adulthood (Arnett, 2000, 2014). Arnett et al. (2014) characterized this period as encompassing instability and unsettledness, especially with respect to work and relationships. They considered that this instability and unsettledness have intensified in the last decades as job markets have become significantly more unstable (Arnett et al., 2014). Entry into the workforce has become more problematic and protracted, and young or emerging, adults remain in education or training for longer periods. These changes and uncertainties are also linked to social factors, such as forming a family later in life—and the level of social support may generally remain low during this period (Pettit et al., 2011; see also von Soest et al., 2020). This all may influence loneliness experiences as well. Furthermore, according to Arnett et al. (2014), all this uncertainty may be linked to higher rates of mental health problems during this life period.

### Depression among women

Alarmingly, incidents of depression are between 1.5 and 3 times higher among women than men (Bromet et al., 2011; Depression: Current Care Guidelines Abstract, 2020; World Health Organization, 2017). According to studies conducted in different countries, this gender difference first emerges during early adolescence, ages 12 to 15 (Hyde et al., 2008; Nolen-Hoeksema & Girgus, 1994; Salk et al., 2017), and continues over the course of life.

Even though the aetiology of gender differences in depression seems to be multifactorial (Hammarström et al., 2009; Harris, 2003; Hyde et al., 2008; Salk et al., 2017), a clear consensus remains lacking regarding explanations for the higher rates of depression in women compared with men, even though different explanatory models have been proposed in relation to gender differences regarding depression. First, much of the research relates to biochemical or biomedical explanatory models governing practice in mainstream psychiatry, where depression is related to biology—especially the reproductive female body—the function of hormones, and neurodevelopmental changes during the pubertal transition (Hammarström et al., 2009; Stoppard, 1997).

A second set of explanations for gender differences in depression originates from sociological theories (Brown & Harris, 1978) and addresses socio-structural circumstances such as the role of poverty, power relations, violence and overall gender inequality, all manifesting as gendered sources of stress and impacting on women’s mental health (Belle & Doucet, 2003; Neitzke, 2016; Stoppard, 1997). To exemplify, girls and women are more likely to experience certain severe life events or major stressors such as violence and sexual abuse than boys and men, which may increase the risk of depression and other types of mental ill health (Harris, 2003; Nolen-Hoeksema & Girgus, 1994).

Finally, psychological models of gender difference state how, behavioural and psychological factors, such as perceptions of competence, greater self-focused attention, dissatisfaction with appearance, and higher rates for anxiety and neuroticism, may explain the gender difference (Hammarström et al., 2009). To exemplify, Bukowski and Adams (2005) proposed that besides girls’ greater exposure to interpersonal stress, their greater consideration of peer likeability and greater affiliative focus in comparison to boys might explain the gender differences in depression.

Importantly, however, these approaches can be criticized because not all women/girls with depression and/or peer difficulties have stressful lives, and not all women/girls who experience violence are depressed (Stoppard, 1997). Moreover, the beforementioned studies remain at the level of everyday phenomena related to gender and social relationships and their links with depression and mental ill health, which enable the essentialization of gender. What greatly affects the above-discussed social realm of women are gender(ed) norms, normative expectations, demands, and related naturalized reiterative acts (Butler, 2004, 2015).

### Associations of loneliness with severe mental illnesses and neuropsychiatric problems

Besides the much-studied topics of loneliness and depression, associations between loneliness and other type of severe long-term mental illness, such as schizophrenia (Nilsson et al., 2008), have been found in earlier studies. Badcock et al. (2015) explored the prevalence of loneliness in psychotic disorders and its associations with psychotic and non-psychotic symptoms and cognitive performance among adults aged 18–64 in Australia. Their findings showed that loneliness was significantly higher among participants with delusional disorders (74.7%) and those with depressive psychosis (93.8%). There have also been studies on neuropsychiatric problems. In a Danish study, no association was found between attention deficit hyperactive disorder (ADHD) and loneliness among adolescent boys in special education (Elmose & Lasgaard, 2017), but an association with Asperger’s syndrome and loneliness was found in an Australian study among both girls and boys (Whitehouse et al., 2009). Contrary to depression, autism spectrum disorders (ASD) and conduct problems, like most developmental disorders, were more commonly diagnosed among boys than girls; the male-to-female ratio averaged at 4:1 in autism but rose to 11:1 in Asperger’s syndrome (Baron-Cohen et al., 2011; Fombonne, 2009). It is possible that this gender discrepancy in ASD reflects the inability of the widely used diagnostic instruments to detect the ways in which Asperger’s syndrome may present in females.

### The present study

We consider it of utmost importance to explore loneliness and mental ill health and their entanglements. These are pressing issues, and while they may entail different, severe presentations among girls/women, boys/men, and non-binary people, many studies (Rich Madsen et al., 2016; Rönkä et al., 2014) show how girls and women seem to experience both loneliness and depression at higher levels in comparison to boys and men. Notwithstanding, some mental issues, such as neuropsychiatric problems, are more common among boys but may be underrecognized among girls and women. This is concerning and may cause further difficulties among girls and women in the absence of timely and appropriate support. Thus, it is important to explore these issues in greater details as well as within gender groups because of the differences in the research challenges in the gender groups. In the present study, we focus on the loneliness and mental ill health entanglements among young adult women.

Overall, the links between loneliness and mental ill health are especially alarming, and when they co-occur, they can seriously distort the daily functioning of young adults transitioning towards adulthood and undermine their educational endeavours, social networking, and career opportunities (Heinrich & Gullone, 2006; Nolen-Hoeksema & Girgus, 1994). They may lead to social exclusion, and in the most severe presentations, both are associated with suicidal behaviour. Furthermore, besides personal suffering, mental ill health causes high healthcare and societal costs for communities (World Health Organization, 2017).

Stoppard (1997) sketched an outline for better understanding women’s mental ill health. This outline accommodates biological, psychological, and social dimensions, and we see it as a useful approach for the present study. Her approach focuses on material–embodied and subjective–discursive aspects of women’s lived experiences regarding their mental ill health. In constructing her theory, she used Harding´s (1986) standpoint theory and approach, which has its origins and is widely used in feminist and gender-responsible research (Harding, 2004; Hesse-Biber, 2012; Louhela, 2019; Smith, 1987).

Standpoint theory posits that the “oppressed location” of women may provide greater insights into the explored phenomenon (Hesse-Biber & Leavy, 2010). We do not propose that women automatically possess a lower position in society, but as the theory emphasizes, we believe that in research, it is of utmost importance to focus on listening to and hearing women’s voices and experiences, especially those with difficult and sensitive experiences (see, e.g., Louhela, 2019). Further, we consider that loneliness and mental ill health are among such experiences and that it is especially important to build a safe space and a trusting atmosphere for these voices to be told, heard and listened to. Thus, we think that qualitative interviews and analysis methods are especially suited to exploring these sensitive and difficult issues (Issakainen & Hänninen, 2016; Ryan-Flood & Gill, 2010).

### Aims and research questions

In the present article, we explore the entanglements of loneliness and different types of mental ill health experiences that may form during the lives of young adult women who have had both negative experiences over their life course. We examine the kinds of factors that contribute to the development of these entanglements. Overall, there is a dearth of research exploring the stories of younger women. A majority of earlier qualitative studies on these topics have been conducted among older adults (see Barg et al., 2006; Lindgren et al., 2014). By the term entanglement, we refer to the different, likely complex, and multiple ways in which loneliness and mental ill health may be interlinked in the study participants’ lives, as described by the participants themselves.

The research questions of the present study are as follows:

- Research question 1: What descriptions of entanglements of loneliness and mental ill health can be detected from the data, as described by women who have experienced both?
- Research question 2: What factors seemed to contribute to the formation of loneliness and mental ill health entanglements, as described by the women?

## Materials and methods

### Northern Finland Birth 1986 as a data source

The participants of this study were from a general population-based Northern Finland Birth Cohort 1986 (NFBC1986). We utilized the NFBC1986 data in our earlier large mixed-methods research project on loneliness (Rönkä, 2017), and the present study is a sub-study for that project. The NFBC1986 is a prospective longitudinal study that comprised 9,432 live-born infants (4,567 girls and 4,865 boys) whose expected date of birth fell between 1 July 1985 and 30 June 1986, who were born in the two northernmost provinces of Finland (Lapland and Oulu). They have been followed in several follow-ups, including the prenatal period, at ages seven (1992–1993) and eight (1993–1994), and at ages 15–16 (2001–2002). The follow-ups included questionnaires for cohort members, their parents, and teachers. Clinical samples were also collected. Different sub-studies have been conducted over the years, and the latest large data collection was conducted in 2019–2020 (Northern Finland Cohorts, 2020).

### Study sample

The study participants both in our earlier research project and in the present study are among those NFBC1986 members who responded to an adolescent questionnaire sent to them when they were 15–16 years old (2001–2002). Altogether, 9,215 cohort members received the questionnaire. It included a one-item self-report on loneliness (“I feel lonely”), with three response alternatives: “being very lonely,” “being sometimes/somewhat lonely,” and “not being lonely at all” during the preceding six months. In total, 7,014 (3,641 girls and 3,373 boys) adolescents responded to the loneliness question in 2001–2002, and among those, 222 (3.2%, 149 girls, 73 boys) responded that they were *very lonely*. In 2013, when they were 27–28 years old young adults, home addresses of those 222 persons were tracked, and an invitation letter was sent to them to participate in an interview for an earlier study project by the authors of the present article. As a few addresses were unknown, the invitation letter was eventually sent to 214 cohort members (144 women and 70 men). Forty responded through a web-link included in the letter or via the first author’s work email. Due to scheduling issues, one interviewee withdrew from the study, and 39 interviews were conducted (32 women, 7 men) by the first author of the present article, in the fall of 2013. The interviewees lived throughout Finland, and they chose the interview setting in their hometowns. Out of 39 interviews, 20 took place in the interviewee’s home, and 14 were conducted in public places, such as a library or a coffee house. Three were done over the phone and two via Skype. All interviews were recorded with professional recording device. The interviews were transcribed verbatim by the first author, resulting in 655 A4 pages of transcriptions. The length of the interviews ranged from 24–93 minutes, and the mean interview length was 57 minutes.

**Figure 1.**
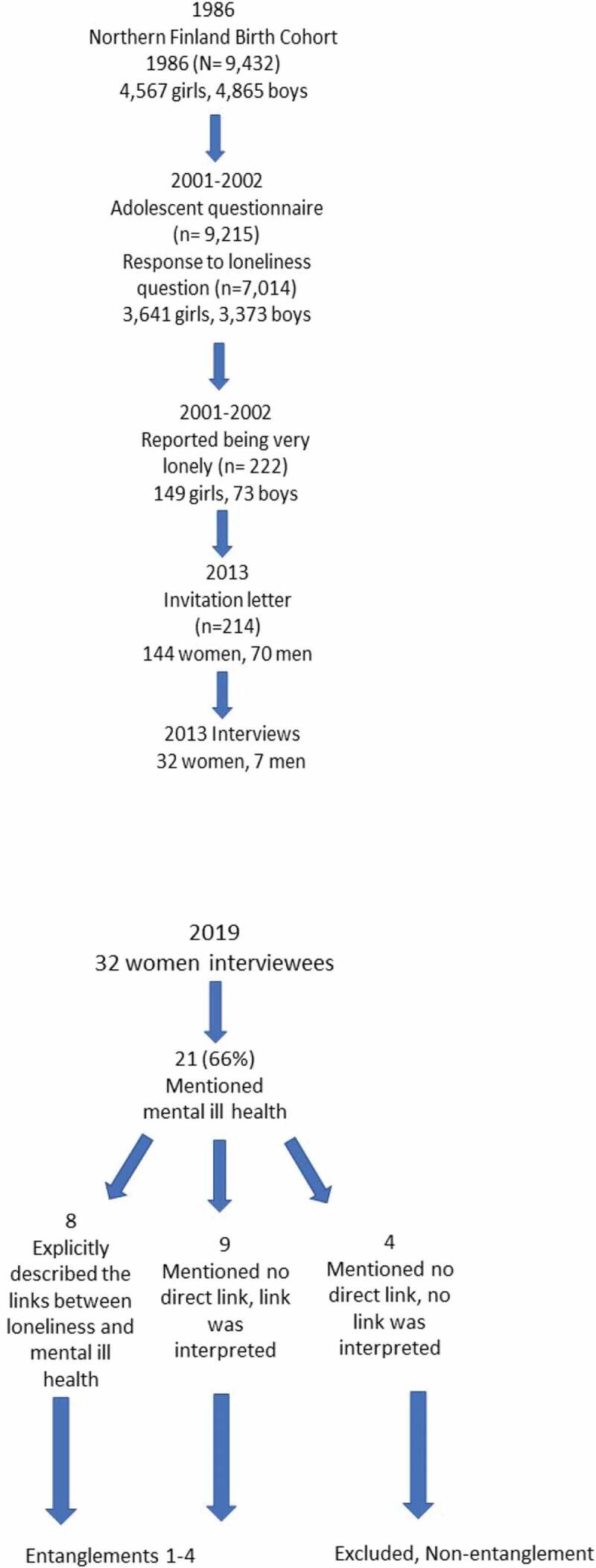
Participants and their selection to the analysis

The Regional Ethics Committee of the Northern Ostrobothnia Hospital District reviewed and approved the NFBC1986 study. When adolescents and parents filled in the questionnaires, written informed consent was obtained from them in 2001–2002. Those adolescents and/or their parents, who refused the usage of their data (or their child´s data), were excluded. Separate ethical approval was requested for conducting the qualitative interview data collection among the cohort members. The Regional Ethics Committee of the Northern Ostrobothnia Hospital District approved the qualitative study in 2013.

(Figure 1 Participants and their selection to the analysis).

### Data production method

For the purpose of our earlier mixed-methods loneliness project (Rönkä, 2017), a semi-structured interview guide was formulated in order to explore the lived experience of loneliness from childhood to young adulthood among the NFBC1986 members who reported being very lonely in their adolescence. The interview themes were formulated based on our earlier findings from three quantitative articles conducted with the adolescent NFBC1986 survey data (Rönkä et al., 2014, 2017, 2013). In these studies, the associations of loneliness and different health and well-being factors were examined. Moreover, selected loneliness theories (Hymel et al., 1999; Weiss, 1973) were utilized to formulate the semi-structured interview guide (Kvale, 2007). In addition to loneliness, the interview guide included other themes closely related with loneliness experience, such as family relations, friendships, belongingness/non-belongingness, school violence and bullying, and health and well-being (see English translation of interview guide in Appendix 1).

In the semi-structured interview method, the interviewee, in interrelation with the interviewer, provides information about the experience of interest and the meaning of that experience for their life. Interviews are seen as the construction site of knowledge (Kvale, 2007) and, based on social-constructivist understanding, knowledge produced in these ways is historical, contextual, and partial (Harding, 1986; Hesse-Biber & Leavy, 2010).

The interviews were planned carefully, and a trusting relationship with the interviewees in the interview situation was formed, as the interviewees openly discussed their experiences and feelings related with the sensitive topics of loneliness, as well as mental ill health (see also (Rönkä, 2017).

### Data analysis methods

According to our findings on our previous qualitative study on loneliness, which was conducted using qualitative theory-driven content analysis^3^ (Rönkä et al., 2018), we noted how strikingly common mental health issues were among research participants, especially women. As it was not possible to further explore the links between loneliness and mental ill health in our previous project, we wanted to focus on the theme of mental ill health, as it relates to loneliness, with greater detail in the present article.

The analysis methods employed in the present study were a form of thematic analysis (Gest et al., 2012). The thematic analysis provides a strategy for organizing and interpreting data to create in-depth understanding that brings together the commonalities and differences in the participants’ descriptions of their experiences. This is done by identifying and generating codes and then clustering them into broader ideas and, finally, related themes (here: different factors related to mental ill health and loneliness in women’s lives). The purpose is to find patterns and their meanings across the data (here, the different ways in which loneliness and mental ill health are entangled) (Crowe et al., 2015). By conducting a thematic analysis, which also include the aspect of time, the aim was to explore the different ways in which loneliness and mental ill health are entangled with each other in women’s lives and the kinds of background factors that seemingly contribute to their formation. Next, the analysis process will be discussed in greater detail (see also Figure 1 on participant selection). The analysis was primarily conducted by the first author (currently a post-doctoral researcher, who, during the time of the interviews was a doctoral student). The second author also reviewed and interpreted the original interview recordings and data. The study findings and their interpretation were constantly discussed by all authors (a docent and university lecturer, and a professor emerita) to achieve mutual understanding and consensus. The second author and third author provided feedback throughout manuscript development.

### Data analysis process

First, the transcriptions of the seven men from the study were excluded, as the focus was on women’s experiences. Then, the transcripts of 32 women were carefully read multiple times in order to get the sense of the whole data. During this reading, all the interviewees who described having had depressive symptomology or other types of mental ill health were identified. Among the 32 women, 21 (66%) described matters related to mental ill health, ranging from depressive moods to diagnosed depression, or to a different type of serious mental emotional disturbance or illness. Over one third of the women had diagnoses of depression or different kinds of serious mental illnesses.^4^ Descriptive codes for different aspects that the women discussed in relation to their mental ill health were generated, including the actual diagnosis or types of mental ill health and the different factors that seemed to be linked to mental ill health. The descriptive codes were written directly to the printed interview transcripts, and a separate file, “a code book” for collecting them to one place was kept. Thereafter, a similar type of coding among these women in relation to their loneliness was performed. The descriptions of the causes for their loneliness, the consequences and emotional aspects of loneliness, as well as the means of loneliness alleviation were coded, and all the codes were added to the code book.

After this coding, the chronological aspects between loneliness and mental ill health were investigated. A chronological description based on the individual stories of these women from childhood to the time of the interview—their young adulthood, was written to a separate word file. Then, a description was conducted from the perspective of loneliness experience with the aim to determine the start of the experience of loneliness and whether it was alleviated or had ended. Following this, the role (i.e., the clustered codes from the thematic analysis regarding mental ill health) of depression or mental ill health was manually “linked and added” to this chronological description. With multiple readings of the interview transcripts it was looked, when depression or other type of mental ill health was first mentioned, when a possible diagnosis was received, its status at the time of the interview, what kind of depression it was, its intensity, and, at a more latent level, the meaning for the participants (Lindgren et al., 2014).

After crafting the stories, specific loneliness–mental ill health entanglements were “revealed” and then examined more carefully. It was noted, that 8 out of 21 women *explicitly talked* about how loneliness evoked depressive feelings or how they thought depression was a consequence of their loneliness. In the remaining 13 interview transcriptions, women did not themselves express such a direct linkage between the two phenomena. Then, yet another round of reading of these women’s transcriptions was conducted. At the same time, the data were read together with the theoretical literature related to both loneliness and mental ill health. It was interpreted that among most of the women (n=9), loneliness and mental ill health experiences seemed to be entangled in different ways. Among the remaining four women, the two did *not* seem to be entangled in any clear way, even with the presence of both loneliness and mental ill health experiences.^5^ Among these women, the severity of both remained at low, and their meaning in the women’s lives seemed more periodic than strong. As the aim of the current study was to look at entanglements, the stories of these four women were excluded from the final round of analysis.

The final round of analysis and interpretation was conducted with the transcripts of the remaining seventeen women, who themselves expressed, or from whom it was interpreted, that loneliness and depression and mental ill health were entangled. Then it was asked, *what kind of* descriptions of entanglements of loneliness and depression were forming. It was explored whether the stories presented any common features, similarities, or differences among the women and the stories were grouped/thematized accordingly. The criteria for grouping were the severity, continuity, and timing of loneliness and mental ill health, background factors of the women, buffering factors, and ways of alleviating the negative experiences. Based on this, four different loneliness–mental ill-health entanglements were constructed from the data.

## Results

The analysis focused on 17 women, whose experiences of loneliness were entangled with different type of mental ill health and mental health problems. Among these women, loneliness and depression fluctuated and recurred over the period between adolescence and young, or emerging adulthood (ages 14–28) instead of being at stable, chronic level at all times, yet some of the women’s experiences had alleviated by the time of the interview. For a majority of the women, especially those who had serious mental illness, their experiences of both loneliness and mental ill health were ongoing.

Based on the analysis of the stories of 17 women, *four types* of entanglements were identified from the data: 1) “Loneliness periodically evoked depressive feelings (three women); 2) “Loneliness and depressive feelings formed an ongoing spiral” (two women); 3) “Loneliness and diagnosed depression/serious emotional disturbance formed periodic spirals” (four women); and the stories of most of the women (eight) belonged to entanglement 4) “Serious emotional disturbance/serious mental illness and loneliness formed an ongoing, difficult spiral.”

(Figure 2)

**Figure 2.**
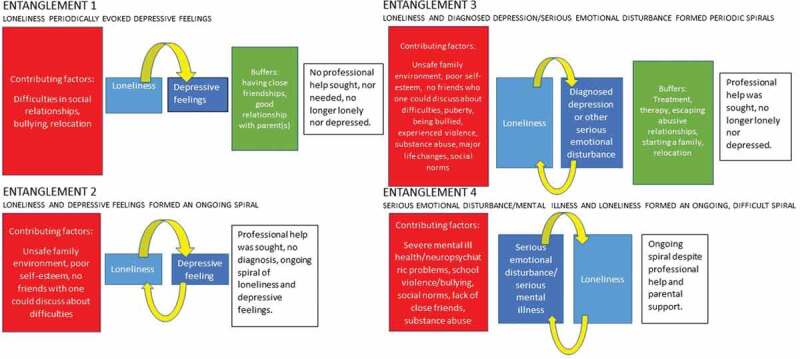
Four types of loneliness-mental ill health entanglements, contributing factors of entanglements, buffers and the mental ill health and loneliness status at the time of the interview

**Loneliness periodically evoking depressive feelings**

The stories of three of the women were grouped under **Entanglement 1**, where *loneliness periodically evoked depressive feelings*. Professional help was not sought for these feelings, and things seemed to resolve on their own (at the time of the interview, they described that they were no longer lonely or depressed). This type of depression that loneliness evoked was not “medical.” As a quote from Kirsi^6^ informs, the two experiences seem to form *a continuum*, ranging from not-so-severe loneliness to a depressive mood:

Loneliness is sadness. That describes loneliness best. Depressed mood, even hopelessness, is related to it; it feels hopeless, this will never end, this is always going to be like this, there is no way out … But then it is no longer only loneliness, it is also, or more like, depression, not medical depression, but other, like low, depressive mood. (Kirsi)

The quote below shows how Tiina’s loneliness evoked depressive feelings and how depression was perceived as *an explicit consequence* of loneliness.

Loneliness felt depressive; it hurt. It is difficult to describe. It was tough, as I had no one to talk to. I believe that is the reason why I had quite heavy depression eventually. (Tiina)

Based on our analysis, loneliness was caused mainly by the experienced difficulties in social relationships. There were periods of insecurity in their friendships, leading to the fear of being left out of social circles. These women had relocated many times in their lives, which made it more difficult to maintain friendships and further influencing loneliness. Also, Tiina, who considered non-medical depression a consequence of her loneliness in the quote above, experienced long-term bullying. She contemplated that bullying led to non-diagnosed social phobia, then to loneliness, and, finally, to non-diagnosed depression. She described that she was not good at maintaining contact with other people, which influenced her loneliness.

However, the depression and loneliness remained at a “manageable level.” Likely buffers against more serious depression or a depression diagnosis seemed to be rather good relationships with their parent(s), and despite problems with their friends, they had close, meaningful friends who they could talk to and confine in about their problems.

### Loneliness and depressive feelings formed an ongoing spiral

In **Entanglement 2**, *loneliness and depressive feelings formed an ongoing spiral*. Then, loneliness was described as a constant *undertone in one’s life*, always recurring and re-surfacing; but it did not turn into an over-encompassing experience for them. The root of their loneliness could be traced to their childhood and an unsafe family environment; they grew up in families marred by alcoholism, which greatly affected their lives. Having an unsafe family environment seemed to contribute negatively to their self-esteem. They described being quiet, shy, and insecure and tended to blame loneliness on themselves. Their parent(s)’ alcoholism caused feelings of shame, and even though they had some close friends, they *were not able* to share with friends their difficulties relating to family life. Thus, in this entanglement, loneliness was caused by an accumulation of difficulties in family life, which in turn evoked strong depressive feelings. These feelings made it difficult to be sociable and approach others, causing further isolation. A continuous spiral—where loneliness reinforced depressive feelings, and depressive feelings reinforced loneliness—was formed. A quote by Tytti exemplifies this spiral:

Tytti: Well, in upper secondary school, absolutely, I was lonely. That was then linked to depression and all that. (—) In loneliness, I was really sad and … mmm … little bit like, empty feeling … mmm, I try to get back to that feeling. I knew it would have been easier if I had someone. I felt like something was missing, something was lacking.

ARR: You had depressive feelings at that time?

Tytti: Yes, they were rather strong. I was not sociable at all, and that it felt bad, but I had no energy or capability of doing anything about it.

Eventually, professional help was sought for depression, but the help from the counsellor or therapist was not seen as useful by these women. Also, they did not receive a diagnosis of depression. At the time of the interview, their loneliness and depression surfaced occasionally.

### Loneliness and diagnosed depression or other serious emotional disturbance formed periodic spirals

The third loneliness and mental ill health entanglement was more intense than Entanglements 1 and 2. These women received a diagnosis and professional help for their depression or other serious emotional illness or disturbance they described having.^7^ However, by the time of the interview, they were no longer lonely or depressed.

Minna experienced two “separate” loneliness–depression entanglements. First, she experienced a phase of severe loneliness in adolescence, which she linked to depression. She first described how her loneliness–depression entanglement had something to do with puberty. There did not seem to be any other reason why she suddenly isolated herself, was not sociable, and was not able to be with or talk to others. However, in the quote below, she describes her feelings during adolescence, when she compared her social situation with that of others and felt inadequacy vis-à-vis her social circles.

It felt like even though I had those couple of close friends, I felt I should have had more. I must have more. Because I knew other people had such big social circles. I guess I did not know back then what kind of person I really was. (Minna)

This reveals implications about social norms related to friendships and the emergence of loneliness: the kinds of social relations one was supposed to have, and if one cannot have these relationships, then loneliness could emerge or intensify. It seemed that during adolescence, the norm for a girl was to have a couple of close best friends. The latter entanglement was “triggered” several years later and linked to major life changes and losses. She started to isolate herself and soon received a depression diagnosis and started medication. She described her depression and loneliness as now under control, but she is scared that the depression will recur. She is also experiencing strong uncertainties about her future. She also ruminated on whether she should start a family, as she was approaching her 30s, which seemed to be the normative age for Finnish women to think about their future from the perspective of a family.

The other women’s stories in **Entanglement 3** were more intense than Minna’s, but they contained some similarities regarding the loneliness–mental ill health entanglement; these two alternated and formed a spiral, which was now resolved. During their school years, these women had very few or no friends; they were very harshly bullied, and one woman also bullied others. School violence made them isolate themselves, and they experienced severe loneliness. Some women described how they turned more inwards in order to protect themselves and cope with everyday difficulties. For instance, they “shut down” in the context of school; they put their headphones on, put hoodies low on their head, and tried to be as invisible as possible. They did not speak or respond to peers. This type of “armour” made them indifferent to the bullying conducted by others. Still, it did not reduce the bullying, and their loneliness and mental ill-being continued. Also, they behaved and dressed differently from their peers and did not follow the normative femininities in their contexts, which was the main reason they were targeted. Furthermore, substance abuse was common among these women, which started at a very young age. Finally, these women described having poor or mentally and physically abusive relationships with their (step)parents. One women later experienced domestic violence. The women felt that they were unable to share their family-related difficulties with anyone else, which intensified their loneliness. All these accumulated adversities formed a negative spiral that was later linked to a variety of mental health issues and, thus, were likely “triggers” for different diagnoses.

However, at the time of the interview, these women revealed that they were no longer lonely and that their mental health issues were under control. Contributory factors and buffers against continuing mental health issues included escaping from abusive relationships, getting therapy, undergoing treatment for substance abuse, and finding a partner and starting a family. Relocation also played a strong role in these stories; relocating with a fresh start contributed positively to their current well-being.

### Serious emotional disturbance or mental illness and loneliness formed an ongoing, difficult spiral

Most of the women in this study belonged to **Entanglement 4**. The severe mental health problems that they experienced seemed to influence their social life and contributed or caused their loneliness. The illnesses they described were Asperger’s syndrome, autism, attention deficit disorder (ADD), ADHD, generalized anxiety disorder, severe depression, borderline personality disorder (BPD), and schizophrenia. Also, one woman had anorexia in her adolescence. Among these women, mental health problems were ongoing, and co-morbidity was common. At the time of the interview, the women were also in treatment.

The women who had an ADHD diagnosis experienced difficulties in their social relationships from early on. They were having difficulties in understanding and acting upon social and behavioural norms in the school context. They were also harshly bullied at school and had no close friends. One woman with these issues described that she acted out violently in school when others were “pushing her buttons,” when they irritated and bullied her. This caused many difficulties for her, and she had to change schools several times over the years. With this type of behaviour, she did not follow the rules and behavioural norms in the context of school, especially those assigned for girls. Also, outside school, some of the women were behaving delinquently, and had substance abuse problems. Furthermore, a couple of women in this Entanglement described being a part of different types of sub-cultures. They had dyed their hair, underwent piercings, later tattoos, and dressed up differently from others. They stood out from the typical adolescent in Northern Finland and did not follow the typical femininities assigned to girls in their contexts. Similar to the previous entanglement, they were targets of school violence, which intensified their loneliness and mental health issues.

Jaana was only recently diagnosed with ADD and Asperger’s syndrome, even though these symptoms had affected much her over the course of her life, causing constant difficulties in multiple ways. In the quote below, she explains how she feels about not being “neurotypical,” the word she uses for those without ADD and Asperger’s syndrome. Not belonging has characterized her whole life, and she feels different from others. All this is intertwined with experiencing loneliness.

It would be nice to be able to have a sense of belongingness with so-called neurotypical people, but our worlds are so different. It is difficult … a sense of non-belongingness has characterized my whole life. I just feel like I do not belong to this world at all, that I am so different, and somehow, I feel like others cannot understand me and I cannot understand others … It feels like I am from a different planet, that I do not belong on this planet. This world is made for a different kind of people. And sort of, how to describe this: The others are like roses, and I am like a thistle. (Jaana)

More seriously, mental health issues entangled with loneliness were linked with suicidal behaviour. One woman was diagnosed with BPD disorder. Loneliness and a sense of not belonging, the latter being a symptom of BPD, has been a constant recurrence in her life. Some years ago, she tried to commit suicide twice.

Based on our interpretation, the severe mental health difficulties in **Entanglement 4** were the main contributors to loneliness and not so much other way around. Also, contrary to the women in Entanglements 2 and 3, these women had parents who supported them and were trying their best to help their children with these difficulties. This has likely been very important for their lives, like the case of the woman with BPD, who described how she could always count on her parents in the most difficult phases of her illness. However, despite the efforts of their parents, their issues continued.

## Discussion

This study explored how loneliness and depression, or other types of mental ill health were entangled with each other through data produced among women in their late 20s, who had experienced prolonged loneliness and mental ill health. By focusing on these entanglements, the study revealed information on different interlinkages between these experiences and their temporality as well as on matters that seemed to contribute to their evolvement among the young adult women. As the main finding of this study, the entanglements between loneliness and mental ill health were grouped into four types based on the similarities and differences regarding their intensity, duration, and the factors contributing to the formation of the entanglements. The findings add important knowledge on the different ways regarding *how* these two negative experiences were entangled with each other over time and show how both difficult experiences may alleviate, at least momentarily. According to the findings, the co-existence of loneliness with different types of mental ill health was strikingly common. As many as 66% of the interviewees talked about their mental health issues. Additionally, more than one-third had a mental health diagnosis. These numbers among lonely women were much higher than, for instance, the prevalence (5.6%) of diagnosed depression disorders in the general adult population in Finland (World Health Organization, 2017). The co-occurrence of neuropsychiatric disorders with loneliness was also high among the women in the present study. Overall, psychiatric comorbidity (having more than one severe mental ill health problem) was high among the research participants (see also Stickley et al. (2017).

### Contributing factors to the formation of entanglements among women

As Figure 2 showed, the accumulation of different life difficulties, mainly an unsafe family environment, the experience of violence, especially at school, and lack of close friends, contributed to the more severe Entanglement 3. In turn, loneliness and mental ill health were alleviated through an escape from abusive social relationships, finding a partner, and starting a family, albeit in combination with treatment or therapy (Entanglement 3). When severe mental ill health was a factor, this caused entanglement to begin, and the difficulties were still ongoing at the time of the interview (Entanglement 4). Nevertheless, when a person had a close relation with a friend, whom she could confide, only depressive feelings, for which professional care was not needed, resulted in loneliness (Entanglements 1 and 2). Overall, the development of depression in Trajectories 1–3 seemed to have strong interpersonal as well as life situational roots. Thus, in general, our findings are in line with the sociological (Stoppard, 1997) explanation for depression among women. Nevertheless, 2 women out of 17 in the present study thought that their depression was likely caused by hormonal changes during their difficult puberty. Thus, a biomedical explanation for women’s depression was also interpreted from the study data (see Stoppard, 1997).

Corroborating earlier studies (Deckers et al., 2017), our findings indicate that difficult loneliness experienced during adolescence may be a risk factor for future mental ill health problems. Earlier longitudinal quantitative studies have shown three to six loneliness trajectories among adolescent, young adult, and elderly populations (Jobe-Shields et al., 2011; see also Rönkä et al., 2018; Wang et al., 2008). Commonly, those who fall into the chronic loneliness trajectory have the most maladjusted profile, including the highest levels of stress, the most depressive symptoms, and the lowest self-esteem of all the trajectories (Vanhalst et al., 2012). Findings from earlier trajectory studies have also shown how difficult life experiences accumulate in the chronic loneliness trajectory (Kamiya et al., 2013; Rönkä et al., 2018). It is important to pay close attention to difficulties relating to adolescents’ loneliness and other socioemotional problems early on in order to prevent their evolvement into difficult spiralling loneliness–mental ill health entanglements.

**Severe mental illness, autism spectrum and neuropsychiatric disorders, and loneliness**

In the most severe loneliness–mental ill health entanglement, Entanglement 4, the women described having other mental illnesses besides depression. The social life of the women was greatly influenced by their diagnoses and were strong causes of loneliness. The loneliness and difficulties in their social relationships further negatively influenced their mental well-being by, for instance, causing them anxiety and distress. Earlier quantitative studies have also found that loneliness is more likely to be a consequence rather than a cause of the symptomology of different types of severe mental illness or psychopathology (Deckers et al., 2017). It was very difficult for those women with severe mental difficulties or neuropsychiatric problems to approach others or to form and maintain social relationships. In general, they had difficulty following social codes and social norms and tired easily in social situations. They did not follow the “social games” that other peers “performed” at school. This contributed to feelings of non-belongingness, a lack of and need for close and meaningful friends, and experienced loneliness.

Contrary to common assumptions, earlier studies on ASD have also shown how young people with ASD are oftentimes aware of their problems with social integration (Attwood, 2000) and report experiencing more loneliness than their “typically” developing peers (White & Roberson-Nay, 2009; Whitehouse et al., 2009). Review of Sumiya et al. (2018) reviewed literature on the associations between ASD and loneliness among children and adolescents and concluded that while young people with ASD had elevated motivation for social interaction, they often failed in their social interaction with peers, felt disappointment, and thereby experienced loneliness. The present study also contained indicators of these mechanisms.

Also, associations between loneliness and ADHD/ADD have been explored in recent studies. Studies have documented that children and adolescents with these symptoms and diagnoses were more likely to experience difficulties in their peer relationships and have few or no friends (Hoza, 2007; Nijmeijer et al., 2008). In the present study, two women with the disorder reflected on how they thought ADHD influenced their social life and intensified their loneliness during school years. Currently, neuropsychiatric disorders as well as ASD remain underdiagnosed and underrecognized among girls and women. This is worrying. Early detection of these issues would help in explaining and understanding their different types of difficulties, especially in the school context, and it would be easier to support these girls before the difficulties accumulate.

### Unsafe family relationships and environments contributing to loneliness-mental ill health entanglement

Besides mental ill health, unsafe family environments characterized by substance abuse, physical and emotional violence, or otherwise difficult relationships with parents, was alarmingly common among the lonely women in this study, and seemed to contribute significantly to loneliness–mental ill health Entanglements 2 and 3. The psychodynamic theory of loneliness, influenced by Freud’s general psychotherapeutic approach, posits that loneliness originates from problems and distortions in early emotional bonds with early attachment figures. Problems with these relationships contribute to inadequate interaction skills and further problems that manifest as difficulties in creating friendships, thus causing loneliness. Earlier studies on mental ill health have also indicated that depression in adolescence may be strongly linked to dysfunctional family relationships and that depressed youth commonly report lower levels of family cohesion and closeness, lower levels of support, more conflicts, more parental control, and poorer communication (Weitkamp et al., 2016). Finally, alcoholism within the family may greatly influence the sense of safety among children of alcoholics, especially with other negative experiences (Anda et al., 2002). As seen in the present study, children in this type of family environment will likely develop difficulties forming a healthy self-esteem and sense of security, which may, in turn, cause further difficulties in different social contexts.

**School violence, loneliness, mental ill health and gendered normativities**

During school years, school violence was a prominent negative experience among almost all the women in this study. A large body of earlier loneliness research has also showed the association between school violence/bullying and loneliness (Acquah et al., 2016 Heiman et al., 2018; Rönkä et al., 2014, 2018). In the present study, experienced school violence was a clear and central cause of loneliness. Being alone in the midst of other people, coupled with experienced mental and physical violence, can be very traumatic

However, we would like to emphasize the role of gender(ed) norms, normativities and related acts, as well as repetition and citation based “doing of gender” as influencing the social realm of women (Butler, 2004, 2015) and the development of the loneliness–mental ill health entanglement—especially in the context of school. Delving deeper, we propose that one of the main reasons that the study participants were targeted for school violence was intolerance of difference as combined with hierarchical use of power in the peer cultures and peer relations in the context of school. In this study we link this to the (hetero)gender(ed) norms and normativities constructed, performed, and repeated (see Butler, 1993, 2004) in adolescent peer cultures (Ringrose & Renold, 2010). That is, arbitrary regulatory normativities are oftentimes constructed in social contexts. In the contexts of the women in the study, it was not suitable for girls to stand out. Dressing differently, occasionally behaving in untraditional ways, not having close friends, and not knowing how to participate in social games did not form part of the normative femininities and were setting them apart from others.

### Buffering factors of loneliness and mental ill-health in women’s lives

Negative social factors were common triggers for loneliness–mental ill health entanglements, but positive social factors were, in turn, important buffers against further difficulties among some of the women. Our findings indicate that in Entanglements 1 and 3, the women had some friends to talk to about their lives and problems. Indications of this were found in previous research conducted among both bullied and lonely youth; having at least one friend may be an important buffer against further emotional or psychological problems (Hodges et al., 1999). Also, in Entanglement 3, the professional help the women received likely buffered against more severe experiences.

Furthermore, loneliness, or simultaneously existing difficult mental ill health, did not disable the formation of social and romantic relationships. In fact, these seemed to be important factors in alleviating loneliness among the women in Entanglement 3. Earlier studies have shown that many women with difficult mental health problems have children and are more likely to marry than men with mental health issues. Indeed, many women had children and partners in this study. Motherhood may offer a normalizing experience for women, providing a new role and meaning to their lives (Nicholson et al., 1998). However, studies on ADHD have shown elevated levels of marital and family problems as well as divorce among adults with ADHD (Biederman et al., 2006; Eakin et al., 2004).

Unlike most of the women in the study, the women in Entanglement 4 had no major issues or bad relationships with their parents. On the contrary, they seemed to have parents who supported them, and devoted much of their time to being there for them. Without their support, their situation might have been much worse. The social support literature offers strong evidence that support from parents is associated with lower levels of depression (Zimmerman et al., 2000). A support network and trustworthy parents are important to achieving positive outcomes, including in terms of keeping children with severe mental illness alive. Still, despite the support of the parents, the loneliness and mental health issues continued at severe level—the findings indicate that besides family support, professional support is also central.

### Strengths and limitations

The strengths of the present study are the usage of the NFBC1986 data, which offered a unique possibility to explore loneliness among young adult women, who themselves had reported being very lonely in in a survey in their adolescence (222 persons out of more than 7,000 reported feeling very lonely in the original survey), and who volunteered at age 27–28 to the interview to discuss about the topic further. The data on these women offered very interesting and important possibilities to explore the entanglements, specifically between loneliness and mental ill health, even though they did not represent a typical group of young, or emerging adults from Northern Finland, or the entirety of lonely young adults. Some of the interviewees also expressed their willingness to help other people—researchers and other young people alike—by participating in the interview. Many of them were also familiar with talking about their problems to psychologists, therapists, or healthcare professionals. This is not necessarily a limitation. Time had passed from the difficult times in their lives, and they now had room to contemplate and discuss their experience in the interview in a very informative way.

Another limitation is that only one interview with each participant was conducted. Multiple interviews could have yielded a more nuanced picture of loneliness. We were expecting to conduct about 20 interviews, but more cohort participant volunteered than expected, and as many as 39 interviewees were finally conducted. As the interviewees lived across the country, with limited resources, only a one-time interview was feasible. However, the participants openly discussed their sensitive experiences in the interviews, and a trusting atmosphere was deemed to have been formed in the interview situations, which can be considered as an important strength of the present study.

## Conclusion

### Conclusive remarks and implications for future research

Unsafe conditions in the childhood home; experienced violence during one’s life, influenced by gender(ed) norms and normativities; and a lack of meaningful social interactions (experiencing the loneliness of emotional isolation in Weiss (1973) terms) were at the heart of the development of loneliness and the ensuing mental ill health (see also Weitkamp et al., 2016). However, in the presence of serious mental illnesses or neuropsychiatric issues, these conditions greatly influenced the respondents’ social life and resulted in loneliness. The four types of loneliness–mental ill health entanglements offer interesting insights into the relationship interlinking difficult life experiences and underlines how both vary over one’s life and how these experiences may be periodic, temporary, or ongoing. A clear, direct causal relationships between the two was difficult to determine, and the metaphor of a spiral was better suited to describing their relationality.

The study underlines how the accumulation of different difficulties may contribute to many socioemotional and mental health problems in a person’s life. The four types of entanglements revealed may be tested in future studies in order to explore whether similar types of entanglements can be found among young people in other contexts and countries and whether these four types help in better understanding and explaining the relationship between loneliness and mental ill health. Furthermore, future studies regarding loneliness and mental health should focus on the gendered aspects of both experiences. For instance, not many studies have focused especially on boys’ or men’s loneliness or depression experiences. Also non-binary gender categories should be included in the research of loneliness and mental ill health, in order to observe the experiences of young people who do not identify themselves within these categories. Furthermore, the methods used for exploring these issues could be more varied and creative, utilizing other data production methods besides interviews.

**Implications for future policy and practice**

The findings of the present study have relevance for social, health, and family policies and practices. According to the findings, social problems and loneliness, both in peer contexts as as problems within families, are pivotal to mental health issues. Even children can experience social and mental health problems. When a child or adolescent experiences loneliness for lengthy periods, for instance, longer than a couple of months, it is prudent to explore the issues behind their loneliness and try to alleviate the experience before it exacerbates.

Adequate government funding of health and social services is important in order to offer possibilities for receiving timely support and mental health treatment. Maternity and child health clinics are also important services regarding early prevention, and professionals working there should be aware of issues related to loneliness and mental health and be able to identify families at risk and offer them low-threshold supportfrom early on. Also, parents and other adults in the lives children and adolescents, such as teachers, should be aware of loneliness and its likely effects. These issues should be discussed in teacher–parent meetings as well as in parental events in early education and comprehensive school.

The present study emphasizes that having different types of severe mental ill health, ASD, or neuropsychiatric issues may be a strong risk factor in developing loneliness. Close attention should be paid to the social realm of young people with these difficulties. These youth may still have a strong need for and sense of belonging (Deckers et al., 2017), and it is important to find ways to support their social relationships and them possibilities for finding meaningful reciprocal social relationships in different contexts, such as at home and school.

Furthermore, tackling school violence is of utmost importance in reducing the loneliness experience, the further negative accumulation of difficulties, and the formation of loneliness–mental ill health entanglements. Whole-school approaches have proven most effective in tackling school violence, but they seem to be most effective in primary school environments (Yeager et al., 2015). Adolescent years are among the most challenging in terms of school violence, loneliness, and the development of loneliness–mental ill health entanglements. More practical programmesfor tackling these issues are needed for middle school years and secondary school environments (grades 7–9 in Finland).

As our findings underlined, traditional gendered norms and normativities (and not following them) contributed to the development of loneliness, school violence, and mental health issues. It is important to deconstruct these detrimental and limiting norms, allow children and adolescents to live up to their full potential, and offer young people possibilities to be themselves. Educational policies and schools should more strongly and comprehensively advocate multidimensional equality within school contexts and offer teachers and parents practical tools for discussing and promoting these issues with pupils in their everyday school life. For instance, arts-based interventions could be useful in promoting empathy and further enhance social and mental well-being

## Notes

1. The quantitative Northern Finland Birth Cohort 1986 (NFBC1986) data used in the present study originally included only two response options for gender: female or male. For this reason, we used the binary category of girls/women and boys/men throughout the present article, which is regrettable. The studies show how, for instance, non-binary adolescents more frequently experience mental health issues in comparison to cis-gender adolescents (Veale et al., 2017). Non-binary gender categories should be included in future research on loneliness and mental ill health. However, the interview data used in the present study also challenge the gender binary, as many participants were critical of normative gender dichotomies and the attendant normative expectations (see Butler, 1993).

2. The gender difference in loneliness ratings is also linked to methodological issues. In survey studies with one-item self-reports, girls report more loneliness in comparison to boys. However, when loneliness is tested using loneliness scales containing multiple items, such as the UCLA loneliness scale (Russell et al., 1978), boys seem to report more loneliness (see, e.g., Junttila & Vauras, 2009).

3. The theory-guided qualitative content analysis of our earlier studies, which was conducted using the same interview data utilized in the present study, has been reported elsewhere (Rönkä, 2017; Rönkä et al., 2018). Then, NVivo10 was utilized for doing the analysis.

4. Four women had a diagnosis of depression, five had depression as well as another type of serious mental illness, and two had other types of serious mental illnesses. The other type of mental illnesses/neuropsychiatric problems described by the women were ASD, ADHD, anorexia nervosa, borderline personality disorder, and schizophrenia. Also, other disorders mentioned were anxiety neurosis and major depressive disorder. According to the mental illness checklist, these conditions may be referred to as serious emotional disturbances (SED) or serious mental illnesses (SMI), as they all belong to diagnostic categories of SED as a condition, which undermines daily functioning at home, school, or in the community (National Institute of Mental Health, 2013). SMI is the term used especially for adults with these diagnoses.

5. For one woman, seeing a school nurse was linked to a difficult break-up with a boyfriend during her high school years, and the situation was characterized by friendship hurdles. It was interpreted that the situation was not causing difficult loneliness for her and that it did not seem to place a strong burden on her mental health. One woman was seeing a psychologist for a short period while attending the University of Applied Sciences, but this was after several years of following a phase of more intense loneliness during elementary school years and grade seven in high school. She did not give much description as to why she saw a psychologist, but she said that it was linked to her personal uncertainties. It was interpreted that this help was not linked to loneliness. One woman described how she had a difficult period in her life, as she had two small children who were not sleeping well. She mentioned this in a maternity clinic and saw a psychologist a couple of times, but she did not find that useful and is now feeling okay. Here, the reason to see a counsellor was not linked to loneliness. Finally, at the time of the interview, one woman was not lonely at all, and the meaning of loneliness was never very strong for her. During her adolescence, she started differentiating herself from her faith, which was a difficult time for her. She went to see a counsellor for difficulties related to religion, but it was interpreted that loneliness was not part of those experiences at the time.

6. All names have been changed.

7. Besides depression, one woman was seeing a psychotherapist due to traumatic events in her life, and another woman had ADHD.

## Geolocation information

Study participants were born in Northernmost parts of Finland; regions of Lapland, Northern Osthrobothnia and Kainuu.

## Appendix

Appendix 1: Semi-structured interview guide

1. Background information

- Name, place of living, current occupational/educational level

2. Educational background and occupation

- Please tell about/describe your educational history and current educational situation.
- Do you have any future educational plans?
- How was your experience attending school? Did you like school? Why or why not?
- Please tell about/describe your occupational history and current occupational situation.

3. Family relations

- What kind of relationship did you have with your family during childhood and adolescence?
- What kind of family relationships do you have now?

4. Friendships

- Please tell about/describe your current friendships.
- What does friendship mean to you? Could you describe your idea of a good friend?
- Have you had any problems in your friendships?

5. Belonging and non-belonging

- Do you feel like you belong to a certain groups now? How about in the past?
- If you do not feel like you belong to any group or have a sense of belonging, why is that?
- Have you ever experienced exclusion? Why was that?

6. School bullying

- Have you experienced being bullied at school? Please describe.
- Does any bullying happen at your work place? Please describe.

7. Loneliness

- Are you experiencing loneliness now? Please describe.
- Please describe what being lonely feels like.
- Did you experience loneliness earlier in your life? Please describe.
- What do you think caused your loneliness?
- Have there been any consequences caused by the loneliness in your life?
- (Have you ever harmed yourself on purpose? This question was typically brought up by the interviewees themselves, but was inquired if not).
- How was your loneliness alleviated?

8. Health and wellbeing

- How would you describe your current life situation?
- How would you describe your current mood?
- How would you describe your current health situation?

9. Future

- What kind of future life plans do you have?

What hopes do you have for the future?
